# Clinical characteristics and outcomes of critically ill COVID-19 patients in Tokyo: a single-center observational study from the first wave

**DOI:** 10.1186/s12879-021-05840-2

**Published:** 2021-02-09

**Authors:** Aya Banno, Toru Hifumi, Hiroshi Okamoto, Minori Masaki, Koichiro Seki, Shutaro Isokawa, Norio Otani, Kuniyoshi Hayashi, Shinichi Ishimatsu

**Affiliations:** 1 Department of Anesthesia and Intensive Care, St. Luke’s International Hospital, 9-1 Akashicho, Chuo-ku, Tokyo, 104-8560 Japan; 2 Department of Emergency and Critical Care Medicine, St. Luke’s International Hospital, Tokyo, Japan; 3 Graduate School of Public Health, St. Luke’s International University, Tokyo, Japan

**Keywords:** Coronavirus disease, COVID-19, ICU, Japan, Mortality, Systemic steroid, Favipiravir

## Abstract

**Background:**

Many studies have been published about critically ill coronavirus disease 2019 (COVID-19) during the early phases of the pandemic but the characteristic or survival of critically ill Japanese patients have not yet been investigated. We sought to investigate the characteristics, inflammatory laboratory finding trends, and outcomes among critically ill Japanese patients who were admitted to the intensive care unit (ICU) with the first wave of COVID-19.

**Methods:**

A retrospective observational study was performed in a single institution in the center of Tokyo. Laboratory-confirmed COVID-19 patients admitted to the ICU from March 19 to April 30, 2020 were included. Trends for significant inflammatory laboratory findings were analyzed. In-hospital death, days of mechanical ventilation or oxygen supplementation, days of ICU or hospital stay were followed until May 26, 2020.

**Results:**

Twenty-four patients were included. Median age was 57.5 years, and 79% were male. The neutrophil-to-lymphocyte ratio was elevated to a median of 10.1 on admission and peaked on Day 10 of illness. Seventeen patients were intubated on Day 11 of illness and received mechanical ventilation. One patient underwent extracorporeal membrane oxygenation. The majority (88%) received systemic steroids, including 16 patients who received high dose methylprednisolone (500–1000 mg). Favipiravir was used in 38% of patients. Two patients, including 1 who refused intensive care, died. Eighteen patients were discharged. Median length of ICU and hospital stay for all patients was 6 and 22 days, respectively. Median length of ventilator dependency was 7 days. Four patients underwent a tracheostomy and received prolonged ventilation for more than 21 days. One patient receiving mechanical ventilation died. All survivors discontinued ventilator use.

**Conclusions:**

Mortality was remarkably low in our single institutional study. Three survivors received mechanical ventilation for more than 3 weeks. Trends of clinically significant laboratory markers reflected the clinical course of COVID-19.

## Background

As of August 1, 2020, 13,163 of 37,778 nationwide coronavirus disease (COVID-19) cases were identified in Tokyo, and 332 (1008 nationwide) patients died [1]. Despite the prolonged exposure to the COVID-19-infected patients that, started from the Chinese New Year, continued through the Diamond Princess cruise ship outbreak, and persisted until the termination of the state of emergency on May 25, 2020, the COVID-19-associated mortality rates in Tokyo have remained remarkably low when compared with those in most Western countries [2–4].

An effective healthcare system, including universal health insurance and the implementation of good personal hygiene practices such as the regular use of masks, are speculated to be the reasons underlying the less severe COVID-19 outcomes in Japan [5]. Furthermore, the level of medical practice and care may have contributed to the less severe outcomes in the Japanese patients; however, the precise reasons for the low mortality rates remain unclear. Thus far, no studies have described the characteristics of the Japanese critically ill COVID-19 patient population who have undergone treatment at domestic hospitals.

We sought to analyze the clinical data of critically ill Japanese patients who were admitted to a general hospital in central Tokyo [6]. This study aimed to describe the clinical characteristics, laboratory trends, and outcomes among patients with the first wave of COVID-19 in an intensive care unit (ICU) setting.

## Methods

### Study design and subjects

This retrospective study enrolled patients with COVID-19 who were consecutively hospitalized in the ICU of St. Luke’s International Hospital, Tokyo, Japan [6] between March 19 and April 30, 2020 [7, 8]. Permission to collect data from electronic medical records were permitted by the hospital and the institutional review board approved this study (approval number 20-R057) and waived the requirement for informed consent in view of the retrospective study design and the urgent need for research insights into this growing pandemic.

The inclusion criteria for this study were ICU admission and laboratory confirmation of COVID-19. Patients with 3 consecutive negative reverse-transcriptase polymerase chain reaction (RT-PCR) test results were excluded from the study. The indications for ICU admission of patients with COVID-19 were respiratory failure that required oxygen supplementation exceeding 5 L/min to maintain a percutaneous pulse saturation (SpO_2_) of more than 94% or the treating physicians’ expectation of progression to further respiratory distress. Patients were admitted to the ICU either directly from the emergency department or transferred from the inpatient wards. Patients designated with a do-not-resuscitate (DNR) order on admission were not admitted to the ICU. The diagnosis of COVID-19 was dependent on positive RT-PCR test results for the severe acute respiratory syndrome coronavirus 2 (SARS-CoV-2) from samples, such as a nasal swab, pharyngeal swab, or sputum [9].

The content of this manuscript has not been published or presented anywhere else, but includes patients who were presented in case reports or case series [7, 8].

### Treatments

Treatment strategies for COVID-19 followed recommendations provided by the National Institutes of Health, Society of Critical Care Medicine, and Japanese Ministry of Health Labor and Welfare [10, 11]. The options for antiviral agents in Japan were favipiravir and lopinavir/ritonavir combination drugs, both were used in clinical trials for treating SARS-CoV-2 infection [12]. Antibiotics and systemic steroids were administered at the discretion of the treating physician. Systemic steroids were administered as either high-dose (pulse therapy of 500–1000 mg methylprednisolone per day for 3 consecutive days) or low-dose (1–2 mg/kg of methylprednisolone for 5–7 days) therapy. Prophylaxis for venous thromboembolism was provided with subcutaneous injections of unfractionated heparin. Supplemental oxygen delivery was maintained by either non-rebreathing mask with a reservoir bag or invasive mechanical ventilation via an endotracheal tube or tracheostomy. An early intubation plan was encouraged. A nasal cannula was worn covered with a surgical mask only for patients who had stabilized after intensive care treatment [13].

### Data collection of variables

We collected information of the baseline characteristics, comorbidities, symptoms, vital signs upon admission, laboratory tests, illness severity during ICU stay, mortality prediction scores (Sequential Organ Failure Assessment [SOFA] score, Acute Physiology and Chronic Health Evaluation [APACHE] II score, Simplified Acute Physiology Score [SAPS II]), ICU therapies (mechanical ventilation, use of vasopressors, renal replacement therapy, enteral feeding), pharmaceutical treatments, complications (arrhythmias, mediastinal emphysema, thromboembolisms), and outcomes. All of the study participants were followed up until May 26, 2020.

### Definitions

The first day of the illness (Day 1) was considered to be the day of symptom onset as reported by the patient. All event dates are presented as days of illness throughout the article. The symptoms at presentation were based on the patients’ complaints and history. Specifically, fever was recorded if the patient claimed to have experienced fever, and this was not based on the initial body temperature measurements. Arrhythmias included new-onset atrial fibrillation, paroxysmal supraventricular tachycardia, and non-sustained ventricular tachycardia. Mediastinal emphysema was diagnosed on chest radiography or chest CT scanning. Thromboembolisms were investigated through ultrasonography and CT scanning.

### Study endpoints

The primary endpoint was death during hospital admission. Secondary endpoints included days of mechanical ventilation, days of oxygen supplementation, days of ICU or hospital stay, and complications (arrhythmias, mediastinal emphysema, thromboembolisms).

### Statistical analysis

Continuous variables are presented as median and interquartile ranges. Categorical variables are reported as the number and percentages. We used the locally weighted scatterplot smoothing (LOWESS) curves to visualize the associations of laboratory findings including white blood cell count (WBC), neutrophil-to-lymphocyte ratio (NLR), C-reactive protein (CRP), and D-dimer with the days of illness. Statistical analysis was conducted with R: A Language and Environment for Statistical Computing, version 4.0.0. (R Foundation for Statistical Computing, Vienna, Austria). The missing data were not imputed or replaced.

## Results

In total, 38 suspected cases of COVID-19, based on patient history and the findings of chest CT imaging, were admitted to the ICU. After confirmation by the results of negative RT-PCR tests, 14 patients were excluded. Thus, 24 patients were included in the final analysis dataset.

### Baseline characteristics

The median age was 57.5 years, and 19 (79%) patients were male. All patients were of Japanese ethnicity. Six patients worked in customer service, 3 were managers, 2 were attorneys, and 1 patient was a health care provider. Baseline characteristics, symptoms, vital signs, and laboratory findings upon admission are listed in Table 1.

**Table 1:** Baseline Characteristics on Hospital Admission

Variable		Range
Age, years	57.5 (49.0, 69.8)	30–85
Male	19 (79)	
BMI, kg/m^2^	24.7 (22.3, 28.2)	19.4–35.4
Underweight (BMI < 18.5)	0 (0)	
Normal (18.5 ≤ BMI < 25)	13 (54)	
Overweight (25 ≤ BMI < 35)	7 (29)	
Obese (35 ≤ BMI)	4 (17)	
Comorbidities		
Hypertension	8 (33)	
Diabetes	7 (29)	
Coronary artery disease	2 (8)	
Asthma	6 (25)	
Chronic obstructive pulmonary disease	3 (13)	
Hyperuricemia	4 (17)	
Malignancy	2 (8)	
Others*	4 (17)	
Current or former smoker	16 (67)	
Habitual or occasional alcohol consumption	11 (58)	
Symptoms		
Fever	23 (96)	
Dyspnea	17 (71)	
Myalgia	4 (17)	
Diarrhea	6 (25)	
Vital signs		
Body temperature†, degrees Celsius	37.7 (37.1, 38.2)	36.2–39.5
Heart rate, beats per minute	98 (94, 115)	66–133
Respiratory rate, per minute	24 (20, 30)	14–44
SpO_2_‡, %	92 (88, 94)	66–97
Laboratory data		
White blood cell count, /mm^3^	7250 (4975, 8950)	3200–19,900
Neutrophil to lymphocyte ratio	10.1 (5.1, 20.1)	2.5–62.0
Platelet count, ×10^4^ /μL	17.4 (15.6, 22.3)	120–389
Lactic acid dehydrogenase, U/L	383 (315, 496)	214–1002
Aspartate aminotransferase, U/L	49.0 (36.5, 68.5)	46.0–97.0
Alanine aminotransferase, U/L	34.0 (27.5, 50.5)	16.0–85.0
Creatinine kinase, U/L	152 (56, 357)	17–2663
C-reactive protein, mg/dL	10.5 (7.4, 16.0)	0.9–33.8
D-dimer, μg/mL	1.2 (1.0, 9.9)	0.5–25.7

*BMI* body mass index, *SpO*_*2*_ percutaneous pulse saturation

Missing data: Habitual or occasional alcohol consumption, 5; SpO_2,_ 5

Data are expressed as numbers (percentage) or median (interquartile range)

*Rheumatoid arthritis, hyperthyroidism, ulcerative colitis, and a past history of pulmonary embolism were present in one patient each, respectively

†Body temperature measurements were all axillary

‡Includes measurements with room air inhalation. Initial SpO_2_ measurements under the influence of oxygen inhalation were excluded

A high NLR was observed, with a median of 10.1. The median platelet count was decreased to 17.4 × 10^4^ /μL, and 6 patients presented with a platelet count below 15.0 × 10^4^ /μL. Four patients presented with CK elevation over 1000 U/L during hospitalization.

### Events and days of illness

The dates of hospital admission ranged from March 11 to April 19, 2020. The median day of illness from the beginning of symptoms to hospital admission was Day 10. Thirteen (54%) were admitted directly to the ICU. In the 11 patients who were admitted to the wards before the ICU admission, the median duration from hospitalization to ICU admission was 6 days. The median day of illness on ICU admission for all patients was Day 11.

### Laboratory findings

**Fig. 1:**
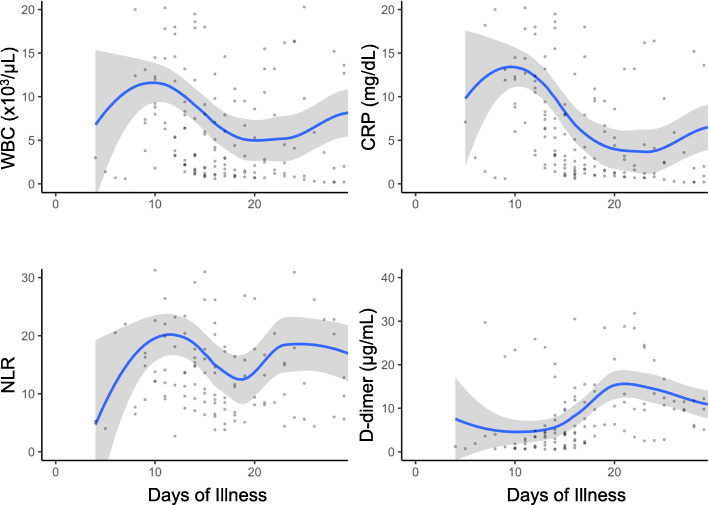
Laboratory Trends According to Days of Illness. WBC: white blood cell, NLR: neutrophil to lymphocyte ratio, CRP: C-reactive protein, LDH: lactic acid dehydrogenase. Locally weighted scatterplot smoothing (LOWESS) curves showing the associations of WBC, CRP, NLR, and D-dimer with days of illness in critically ill COVID-19 patients. The shaded area represents the 95% confidence intervals. Each patients’ laboratory tests were obtained on the day of admission but are plotted according to the day of illness

The associations of WBC, CRP, NLR, and D-dimer measurements with days of illness for all patients are shown in Fig. 1. WBC, NLR, and CRP followed a similar trend and peaked on Day 10 of illness. LDH was elevated in the initial phase of illness and subsequently decreased. D-dimer was more likely to increase after Day 20 of illness.

### ICU admission and therapy

**Fig. 2:**
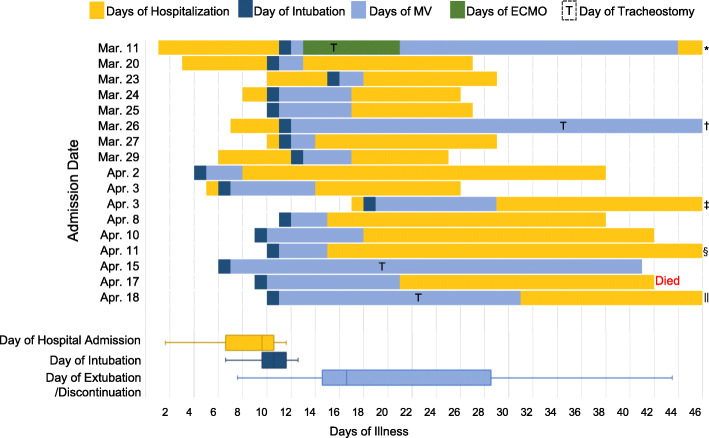
Clinical Courses and Outcomes of Patients that Received Invasive Mechanical Ventilation. MV: mechanical ventilation, ECMO: extracorporeal membrane oxygenation. The results of all patients were followed up and updated on May 26, 2020. The clinical course of patients is illustrated in chronological order by the date of admission. Eighteen patients were discharged. One patient received extracorporeal membrane oxygenation treatment. Thirteen patients were successfully extubated. Among 4 patients who underwent tracheostomy, 3 were successfully weaned off of mechanical ventilation, and 1 patient died. The median days of illness on hospital admission, intubation, and extubation/discontinuation of mechanical ventilation were 10, 11, and 17 days, respectively, and are displayed in the box and whisker plot in yellow, navy and blue. *Still hospitalized, †Discontinuation of mechanical ventilation on Day 51 and still hospitalized, ‡Discharged on Day 47, §Discharged on Day 51, ||Still hospitalized

Severity and mortality prediction scores, therapies, and pharmaceutical treatments in the ICU are described in Table 2. Seventeen (71%) patients were intubated and received mechanical ventilation at a median of Day 11 of illness. For the patients who received mechanical ventilation, the lowest arterial partial pressure of oxygen to fraction of inspired oxygen (PaO_2_/F_I_O_2_) ratios after intubation showed a median value of 169. Four patients who underwent prone positioning had nadir PaO_2_/F_I_O_2_ ratios from 83 to 150. Tracheostomy was performed on Days 5, 13, 14, and 22 of illness in 4 patients (Fig. 2).

**Table 2:** Severity and Mortality Prediction Scores, ICU Therapies, and Pharmaceutical Treatments

Variable	
Scoring on ICU admission	
SOFA score	6 (3, 7)
APACHE-II score	17 (13, 19)
SAPS-II score	31 (21, 38)
ICU therapy	
Invasive mechanical ventilation	17 (71)
Days of illness on intubation*, days	11 (10, 12)
Nadir PaO_2_/F_I_O_2_ ratio*	169 (108, 183)
Maximum positive end-expiratory pressure*	12 (10, 12)
Prone positioning*	4 (24)
Neuromuscular blockade*	2 (12)
Extracorporeal membrane oxygenation*	1 (6)
Inhaled nitrate oxide*	1 (6)
Tracheostomy*	4 (24)
Vasopressors	15 (63)
Renal replacement therapy	2 (8)
Enteral feeding within 24 h of ICU admission	16 (67)
Enteral feeding within 48 h of ICU admission	19 (79)
Pharmaceutical Treatments	
Systemic steroids	
High-dose methylprednisolone	16 (67)
Low-dose methylprednisolone	5 (21)
No systemic steroid	3 (13)
Antiviral agents	
Favipiravir	9 (38)
Lopinavir/Ritonavir	4 (17)
Antibacterial agents^†^	
Piperacillin/Tazobactam and Azithromycin	7 (29)
Piperacillin/Tazobactam and Levofloxacin	4 (17)
Ceftriaxone and Azithromycin	10 (42)
Ceftriaxone and Levofloxacin	1 (4)
Ceftriaxone and Minocycline	1 (4)
Ampicillin/Sulbactam and Azithromycin	1 (4)
Anticoagulants	
Subcutaneous bolus injection of unfractionated heparin	6 (25)
Intravenous continuous unfractionated heparin	12 (50)
No anticoagulant	6 (25)

*SOFA* Sequential Organ Failure Assessment, *APACHE* Acute Physiology and Chronic Health Evaluation, *SAPS* Simplified Acute Physiology Score, *PaO*_*2*_*/F*_*I*_*O*_*2*_ partial oxygen pressure/fraction inspired oxygen. Missing data: SAPS-II, 2

Data are expressed as numbers (percentage), or median (interquartile range)

* Measurements and treatments in mechanically ventilated patients are displayed

†Initial combination of antibiotics use for the whole hospitalization course are shown

Continuous injections of vasopressors were administered in 13 patients who underwent mechanical ventilation and 2 who did not. Two patients without previous kidney disease received renal replacement therapy for uremia and hyperkalemia, respectively.

Systemic steroids were administered in 21 (88%) patients. Favipiravir and lopinavir/ritonavir were administered in 9 (38%) and 4 (17%) patients, respectively. Subcutaneous injections of unfractionated heparin were administered in 12 (50%) patients as prophylaxis for venous thromboembolism, and 6 (25%) patients received continuous intravenous unfractionated heparin for evident thromboembolisms or arterial fibrillation.

### Outcomes

**Fig. 3:**
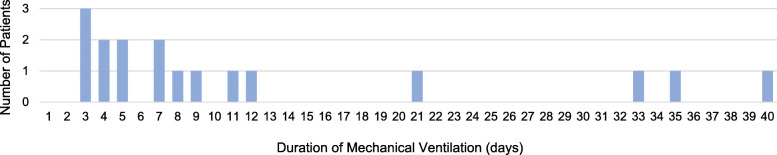
Histogram Displaying the Number of Patients According to Duration of Dependence of Mechanical Ventilation. Median duration of ventilator dependency was 7 (range, 3–40) days. Patients ventilated for between 3 and 12 days were successfully extubated. Four patients received prolonged mechanical ventilation and underwent tracheostomy

In this study population of 24 patients, 2 (8%) died. One patient was designated with a DNR order after ICU admission and died within 24 h of entry. In total, 18 patients were confirmed to have been discharged and had returned home. Four patients remain hospitalized. The median lengths of ICU and hospital stay were 6 and 22 days, respectively. With regard to patients who received invasive mechanical ventilation, the median duration of ventilator dependence was 7 days. Thirteen patients were successfully extubated. Among the 4 patients who underwent tracheostomy, 3 were successfully weaned off from the mechanical ventilator, and 1 patient died. Figure 2 illustrates the clinical course of patients who received invasive mechanical ventilation. The association between hospitalization or mechanical ventilation duration and days of illness is displayed in the chronological order of hospitalization based on the patients’ admission dates. The distribution of the duration of mechanical ventilation is shown in Fig. 3. Supplemental oxygen was administered in all patients. The median duration of oxygen supplementation was 13 days. The incidence of complications, including arrhythmia, mediastinal emphysema, and thromboembolisms, is listed in Table 3.

**Table 3:** Outcomes of Critically Ill COVID-19 Patients

Variable	
Total deaths	2 (8)
Discharged	18 (75)
Still hospitalized	4 (17)
Length of stay	
In ICU, days	6 (4, 13)
In hospital, days	22 (19, 31)
Invasive mechanical ventilation relating outcomes	
Extubated*	13 (76.5)
Tracheostomy with discontinuation of mechanical ventilator*	3 (18)
Death*	1 (6)
Days of oxygen supplementation, days	13 (9, 21)
Complications	
Arrhythmia†	7 (29)
Mediastinal emphysema‡	3 (13)
Thromboembolisms§	4 (17)

*ICU* intensive care unit. Data are expressed as numbers (percentage), or median (interquartile range)

* Variables in patients receiving invasive mechanical ventilation

†Includes four new onset arterial fibrillation, two paroxysmal supraventricular tachycardia, and one non-sustained ventricular tachycardia

‡Mediastinal emphysema was diagnosed prior to endotracheal intubation in one patient and during mechanical ventilation in two patients with radiological findings

§Deep vein thromboembolisms were evident in three patients with ultrasound investigations. One other patient developed multifocal cerebral embolisms

## Discussion

This study evaluated the mortality rate and clinical outcomes in 24 critically ill Japanese patients with COVID-19 who were admitted to the ICU between late March and mid-April 2020. The majority of patients were male, most were normal weight, and two-thirds were former or current smokers. Mortality rates until follow up in the current study were 8% for overall ICU admissions and 6% for mechanically ventilated patients.

The highlight of this study was its focus on a population in East Asia besides China, which was the initial epicenter of COVID-19. East Asia is reported to have a lower COVID-19 mortality than that in the American or the European countries [14]. As of July 1, 2020 we were unable to find published reports of critically ill patients in East Asia, and the reasons underpinning these regional differences are unclear. The mortality rate was low in our study, including survivors who underwent prolonged mechanical ventilation for more than 3 weeks. The clinical course of patients who were intubated was described based on days of illness in this study. Furthermore, we reported the trends of laboratory findings such as NLR, CRP, and D-dimer throughout the disease course. The use of the antiviral agents of favipiravir and lopinavir/ritonavir and the high frequency of systemic steroid administration may have been practices that are unique to Japan and requires further assessment and documentation.

In this study, 17 of the 24 patients received mechanical ventilation for less than 2 weeks, with the exception of 4 patients that required tracheostomy and prolonged ventilation. The intubation rate of 71% in the ICU setting was lower than the 88% reported in a multicenter study in Italy [4] and 93% reported in a large single institutional study in New York, [15] but was similar to the rates of 71 to 79% reported in multiple multicenter studies in the United States [16–18] and was higher than the rates of 30 to 47% reported during the early phase of the endemic in Wuhan, China [19–21]. The median PaO_2_/F_I_O_2_ ratio of the intubated patients was 169, which was close to the value of 160 in the Italian study [4] although higher than that reported in most American studies [16, 18]. The median duration of mechanical ventilation was 7 days, and the extubation rate was 77%. The 8% mortality rate in the current study was significantly lower than the rates of 26 to 78% reported in other affected cities or regions [4, 15–18, 20, 22, 23].

An early intubation strategy was enforced, and the decision to adopt invasive mechanical ventilation was made promptly. Thresholds for intubation were lowered to avoid the use of high-flow nasal-cannula or non-invasive positive pressure ventilation therapy, which carry the risk of aerosol contamination that may increase the SARS-CoV-2 transmission risk for health care providers [13]. Patients who were at risk were closely monitored and transferred to the ICU without delay. This practice may explain the relatively low SOFA, APACHE-II, and SAPS-II scores on ICU admission. Unlike in the Lombardy Region or New York, [4, 16] Tokyo did not experience a severe outbreak of COVID-19. Therefore, the ICU capacity and the need for ventilators were not overwhelmed. These factors may have permitted early intervention before the point-of-no-return deterioration or death.

The median BMI of 24.7 kg/m^2^ in our study was significantly lower than the mean of 30 kg/m^2^ reported in New York [15, 16] although it was similar to that of Chinese patients [24]. According to previous studies, obesity has been associated with worse outcomes [25, 26]. The lower BMI of our study subjects may have been protective. Further investigations are required to understand the influence of body weight on outcomes in Japanese patients.

Elucidating the underlying inflammatory mechanism of COVID-19 may aid clinicians’ comprehension of the disease and clarify the deterioration in clinical course on Day 10 of illness as well as trends of laboratory findings [9, 27]. In our study, the median day of illness on ICU admission and intubation was Day 11. Laboratory findings such as WBC, NLR, and CRP consistently peaked at this phase. NLR and CRP are well-known indicators of inflammatory damage and have been associated with worse outcomes [20, 28, 29]. However, changes in laboratory findings throughout the clinical course of the disease have not been delineated. Combined assessment of the clinical timeframe and laboratory trends may be beneficial for predicting patients’ course of COVID-19. Furthermore, coagulation abnormalities are predominant features of COVID-19, and high D-dimer levels are reported on initial laboratory testing [30]. In this study, D-dimer peaked on Day 20 of illness. Recognition of these changes guided the institutional clinical practice for active surveillance for the detection of thromboembolism and early administration of prophylaxis [31].

Corticosteroid administration in critically ill patients with COVID-19 remains controversial [32, 33]. In previous studies, the use of systemic steroids in ICU patients varied from 26 to 50% [15, 16, 20, 22]. Low dose steroid treatment in the range of 1 to 2 mg/kg were frequently administered [32, 34]. In this study, 88% of patients used systemic steroids, including 16 survivors that received high dosage (500 to 1000 mg) of methylprednisolone. Given the underlying inflammatory mechanisms of COVID-19 [35, 36] and the trend of inflammatory laboratory findings, systemic steroids may play a role in the suppression of inflammatory responses when used judiciously [8]. Further studies are necessary to evaluate the efficacy of systemic steroid therapy in COVID-19.

Favipiravir, a flu drug developed in Japan [37] was the main antiviral agent predominantly used during the observation period, after April 1, 2020.

Because favipiravir was used in only 10 critically ill COVID-19 patients in the current study, further investigation and the results of clinical trials are anticipated [38].

### Limitations

Several limitations of this study need to be addressed. First, given that this was a single-center retrospective cohort study, there was potential selection bias including the inclusion criteria of our subjects. Moreover, uncontrolled confounding factors may have been present. Second, the number of patients included in this study was low, therefore we could not align characteristics or treatment options among the study subjects. Third, statistical analyses were not conducted due to the small sample size.

## Conclusions

Frontline institutions in Tokyo faced prolonged exposure to COVID-19. However, the mortality rate among Japanese patients with COVID-19 was remarkably low. Furthermore, there were survivors among patients who received prolonged mechanical ventilation. The identification of laboratory trends throughout the clinical course of COVID-19 may aid comprehension of the pathophysiology of COVID-19 and facilitate optimal treatment of critically ill patients with COVID-19. Although the sample size of our study was small, this study withholds not only scientific implications but also documentary significance of the first wave of the COVID-19 pandemic in Japan.

## Data Availability

The datasets generated and analyzed during the current study are not publicly available due to them containing information that could compromise individual privacy but are available from the corresponding author on reasonable request.

## Abbreviations

APACHE: Acute physiology and chronic health evaluation
BMI: Body-mass-index
CK: Creatine kinase
COVID-19: Coronavirus disease 2019
CRP: C-reactive protein
CT: Computed tomography
DNR: Do-not-resuscitate
ICU: Intensive care unit
LDH: Lactic acid dehydrogenase
LOWESS: locally weighted scatterplot smoothing
NLR: Neutrophil to lymphocyte ratio
PaO_2_/F_I_O_2_: Arterial partial pressure of oxygen to fraction of inspired oxygen
RT-PCR: Reverse-transcriptase polymerase chain reaction
SAPS: Simplified acute physiology score
SARS-CoV-2: Severe acute respiratory syndrome-coronavirus-2
SOFA: Sequential organ failure assessment
SpO_2_: Percutaneous pulse saturation
WBC: White blood cell
